# Supplementary material for: Preschool Language Development of Children Born to Women with an Opioid Use Disorder
Source: Children (Basel). 2021 Mar 31;8(4):268. doi: 10.3390/children8040268 (PMC8066299; doi:10.3390/children8040268)
Supplement: Supplementary file 1 [file children-08-00268-s001.pdf]

Supplementary

Table S1: Customized Child Rearing Environment Questionnaire – Adapted from the Dunedin Multidisciplinary Health and Development Study.

No.	Item
1	Go to the park, playground, or library
2	Spend time talking to you about things
3	Be read a story
4	Go shopping or to the supermarket
5	Share a family meal (i.e., mum, dad, kids)
6	Visit relatives or friends who have children
7	Learn a new skill from you (e.g., how to stack blocks, kick balls)
8	Help with something you are doing (e.g., baking, cleaning)
9	Play a game with you (e.g., puzzles, peekaboos, ball)
10	Get out of the house with parent or older person
11	Go to beach or pool
12	Visit animal park, zoo or farm
13	Make things (e.g., craftwork, coloring)
14	Get book from library
15	Go on a family outing (e.g., movies, picnic, sport)
16	Go to doctors or clinic
17	Engage in messy play
18	Stay overnight with friends or relatives
19	Friend to play
20	Go to a friend's house
21	Be cared for by other parent
22	Spend time playing with other parent

All item responses in a Likert-type scale with responses from 0 to 4: 0: "never"; 1: "1-3 months"; 2: "monthly"; 3: "weekly"; 4: "daily".
